# Severe periodontitis patients with well-controlled type 2 diabetes display a distinct subgingival microbiome with increased Saccharibacteria compared to systemically healthy controls

**DOI:** 10.3389/fcimb.2026.1814983

**Published:** 2026-03-30

**Authors:** Shiqing Shi, Shaoying Wang, Shiying Li, Ruifang Lu, Shaoxia Pan, Feng Chen, Xuesong He

**Affiliations:** 1 Department of Prosthodontics, Peking University School and Hospital of Stomatology & National Center for Stomatology & National Clinical Research Center for Oral Diseases & National Engineering Research Center of Oral Biomaterials and Digital Medical Devices & Beijing Key Laboratory of Digital Stomatology & National Health Commission Key Laboratory of Digital Stomatology & National Medical Products Administration Key Laboratory for Dental Materials, Beijing, China; 2 Central Laboratory, Peking University School and Hospital of Stomatology & National Center for Stomatology & National Clinical Research Center for Oral Diseases & National Engineering Research Center of Oral Biomaterials and Digital Medical Devices & Beijing Key Laboratory of Digital Stomatology & National Health Commission Key Laboratory of Digital Stomatology & National Medical Products Administration Key Laboratory for Dental Materials, Beijing, China; 3 Second Clinical Division, Peking University School and Hospital of Stomatology & National Center for Stomatology & National Clinical Research Center for Oral Diseases & National Engineering Research Center of Oral Biomaterials and Digital Medical Devices & Beijing Key Laboratory of Digital Stomatology & National Health Commission Key Laboratory of Digital Stomatology & National Medical Products Administration Key Laboratory for Dental Materials, Beijing, China; 4 Department of Periodontology, Peking University School and Hospital of Stomatology & National Center for Stomatology & National Clinical Research Center for Oral Diseases & National Engineering Research Center of Oral Biomaterials and Digital Medical Devices & Beijing Key Laboratory of Digital Stomatology & National Health Commission Key Laboratory of Digital Stomatology & National Medical Products Administration Key Laboratory for Dental Materials, Beijing, China; 5 Department of Microbiology, American Dental Association Forsyth Institute, Somerville, MA, United States

**Keywords:** next-generation sequencing, Saccharibacteria, severe periodontitis, subgingival microbiome, type 2 diabetes mellitus

## Abstract

**Introduction:**

Type 2 diabetes mellitus (T2DM) is a major systemic risk factor that exacerbates periodontitis, with microbial dysbiosis recognized as an important mechanism. However, whether a well-controlled diabetic state still exerts a distinct influence on the subgingival microbiome remains to be fully elucidated.

**Methods:**

This study compared the subgingival microbiota composition in patients with generalized Stage III/IV periodontitis, categorized into a systemically healthy Control group (n = 30) and a well-controlled T2DM group (HbA1c < 8%, n = 30). Subgingival plaque samples were collected using curettes from the deepest diseased sites. The V4 hypervariable region of the 16S rRNA gene was sequenced using Illumina NovaSeq 6000 platform.

**Results:**

Demographic characteristics and periodontal parameters were comparable between groups, except for glycemic indices. Alpha and beta diversity analyses demonstrated no significant differences in overall microbial diversity or community structure (ANOSIM, *P* > 0.05). However, the T2DM group exhibited a distinct diabetic-associated microbial signature. The T2DM group showed a significant enrichment of the phylum Saccharibacteria (formerly TM7), particularly *Nanosynbacter lyticus*. In contrast, the phylum Actinomycetota, predominantly represented by the genus *Actinomyces*, was significantly reduced in the T2DM group. Notably, classical “Red Complex” pathogens were not identified as discriminative biomarkers between the groups. Additionally, correlation analysis revealed that Saccharibacteria abundance was positively associated with HbA1c and fasting blood glucose levels.

**Discussion:**

These findings demonstrate that even with adequate glycemic control, the diabetic microenvironment exerts a unique selective pressure on the subgingival microbiome, favoring the expansion of specific epibiotic bacteria like Saccharibacteria while reducing commensal *Actinomyces*.

## 1 Introduction

Periodontitis is a chronic, multifactorial inflammatory disease characterized by the progressive destruction of the tooth-supporting apparatus (Papapanou et al., 2018). Clinically manifested by gingival bleeding, pocket formation, and alveolar bone resorption, it is a leading cause of tooth loss in adults and poses a significant global public health challenge, with severe periodontitis affecting approximately 12.50% of the global population (Liccardo et al., 2019; Nascimento et al., 2024). The pathogenesis of periodontitis involves a complex interplay between subgingival microbial dysbiosis and the host immune-inflammatory response (Hajishengallis and Lamont, 2012; Abdulkareem et al., 2023). While the “Red Complex” bacteria, Porphyromonas gingivalis, Tannerella forsythia, and Treponema denticola, have traditionally been viewed as keystone pathogens (Socransky et al., 1998), current paradigms increasingly emphasize the critical role of host systemic factors in modulating the oral microbiome and disease trajectory (Hajishengallis, 2015; Tonetti et al., 2018). Among these, the bidirectional relationship between Type 2 Diabetes Mellitus (T2DM) and periodontitis is well established (Genco and Borgnakke, 2013; Belstrøm, 2020; Herrera et al., 2023). Patients with T2DM typically exhibit exacerbated periodontal clinical parameters and a higher risk of severe periodontitis compared to systemic healthy individuals (Stöhr et al., 2021; Alshihayb et al., 2021). Furthermore, the glycemic control condition is tightly associated with both disease progression and treatment outcomes (Demmer et al., 2012; Mizutani et al., 2024), underscoring the importance of the metabolic environment in periodontal pathophysiology.

Despite this recognized association, consensus regarding the specific nature of T2DM-associated subgingival dysbiosis remains fragmented. While some studies have reported a distinct pro-inflammatory microbial profile enriched with classical periodontal pathogens in diabetic individuals (Shi et al., 2020; Balmasova et al., 2021), others have observed a reduction in traditional pathogens accompanied by an enrichment of poorly characterized or low-abundance taxa (Casarin et al., 2013; Ganesan et al., 2017). These discrepancies may largely stem from methodological limitations in earlier studies. First, the reliance on low-throughput techniques, such as 16S rRNA gene cloning and sequencing, limited the ability to capture the full complexity of the subgingival microbiota, particularly low-abundance or novel taxa (Mardis, 2008; Metzker,2010). Second, high heterogeneity in the glycemic control of study cohorts acts as a major confounding factor (Yang et al., 2020). Severe hyperglycemia and the resulting systemic inflammation impose a strong, non-specific selective pressure on the subgingival microbiota, potentially masking more subtle, diabetes-related ecological shifts intrinsic to the condition (Duarte et al., 2014; Miranda et al., 2017; Bolchis et al., 2025).

To address these limitations, minimizing the confounding effects of extreme metabolic imbalance is essential. Investigating well-controlled T2DM patients allows us to assess the diabetes-associated microbial signatures that persist even when clinical glycemic targets are met. Identifying such persistent microbial signatures is clinically relevant, as they may help understand the suboptimal response to periodontal treatment frequently observed in patients with T2DM (Llambés, 2015; Belizário et al., 2024). Furthermore, compared with paper point sampling, subgingival curettage enables the collection of adherent, structurally organized biofilms that more accurately reflect the pathogenic microbial communities driving periodontal destruction (Beyer et al., 2017). When combined with high-throughput next-generation sequencing (NGS), this approach allows for a high-resolution characterization of biofilm composition and structure (Griffen et al., 2012).

Therefore, this study aims to characterize the subgingival microbiome associated with severe periodontitis (Stage III/IV) in Chinese patients who were either systemically healthy or with well-controlled T2DM. By employing high-resolution 16S rRNA gene sequencing on subgingival samples collected using curettage, we sought to identify diabetes-associated subgingival microbial signatures in severe periodontitis and to provide insight into the potential mechanistic link between systemic metabolic disorder and periodontal pathogenesis.

## 2 Materials and methods

### 2.1 Participant selection and grouping

This cross-sectional study was performed according to the Declaration of Helsinki. The study protocol was approved by the Ethics Committee of Peking University School of Stomatology (Approval Number: PKUSSIRB-2024105210) and registered with the Chinese Clinical Trial Registry (Registration Number: ChiCTR2500109048). All participants signed an informed consent form after a thorough explanation of the study’s purpose and procedures.

To ensure the reliability of the experimental findings, a scientifically rigorous sample size estimation was performed prior to patient enrollment. Microbiome data are inherently high-dimensional and have substantial inter-individual heterogeneity. In accordance with established statistical strategies for microbiome research (Casals-Pascual et al., 2020), the sample size calculation in this study was primarily based on the Shannon index. Based on our preliminary data, the Shannon index was 5.8 ± 0.6 for the T2DM group and 5.4 ± 0.4 for the control group. The statistical parameters were established *a priori* with a two-tailed significance level (α) of 0.05, a statistical power (1 - β) of 0.80, and an allocation ratio of 1:1. Utilizing G*Power software (Version 3.1) based on an independent samples t-test model, the minimum required sample size was calculated to be 26 subjects per group. Accounting for an estimated 15% attrition rate, the final sample size was prospectively set to 30 subjects per group.

A total of 60 participants were enrolled and categorized into two groups: a T2DM group (n = 30) and a systemically healthy Control group (n = 30). All participants were diagnosed with generalized periodontitis (Stage III/IV, Grade A/B) according to the 2017 World Workshop on the Classification of Periodontal and Peri-Implant Diseases and Conditions (Papapanou et al., 2018).

Inclusion criteria were as follows: (1) Diagnosis of chronic periodontitis (Stage III/IV, Grade A/B); (2) For the T2DM group: a confirmed diagnosis for ≥ 3 years, well-controlled via conventional hypoglycemic medication (e.g., metformin or insulin), and glycated hemoglobin (HbA1c) levels < 8%. This specific threshold was adopted in accordance with the individualized targets recommended by the ACP guidance (Qaseem et al., 2018), which advocate for less stringent goals in older patients with prolonged disease durations to minimize disease risks. Exclusion criteria were as follows: (1) history of non-surgical or surgical periodontal therapy within the preceding 6 months; (2) use of systemic or local antibiotics or anti-inflammatory medications within the preceding 3 months; (3) smoking; (4) pregnancy or lactation; (5) presence of acute or severe chronic diabetic complications, including but not limited to diabetic retinopathy, nephropathy, or cardiovascular disease; (6) presence of oral mucosal lesions or malignancies; (7) history of head and neck radiotherapy or chemotherapy within the past 5 years; (8) physical or cognitive conditions that could impair oral hygiene practices or adherence to the study protocol.

**Figure 1:**
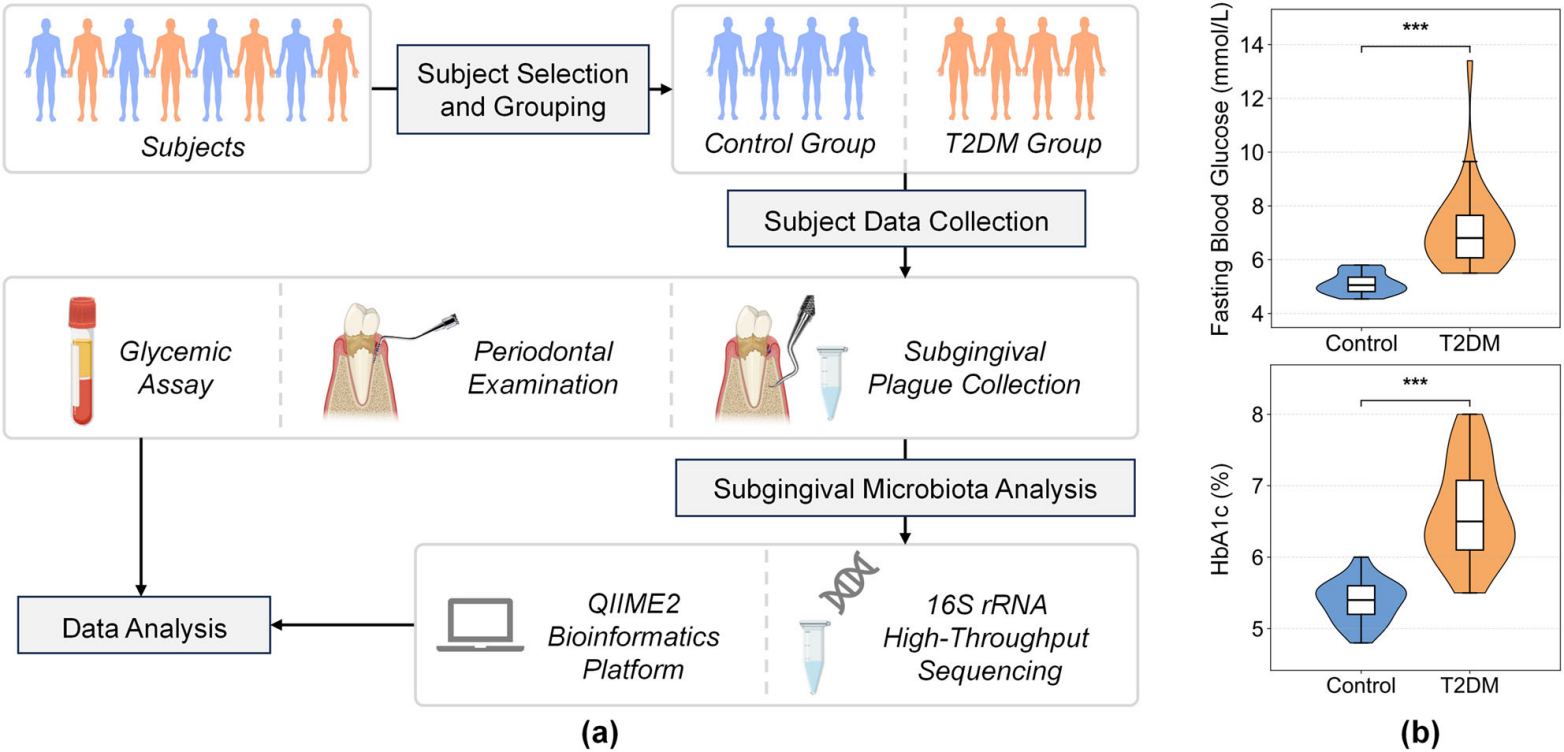
**(a)** The flowchart of the study design. **(b)** Comparison of blood glucose levels between the two groups. Significance levels are indicated as follows: ^∗∗∗^*P* < 0.001.

Demographic data and clinical variables, including duration of diabetes, fasting blood glucose (FBG), HbA1c levels and periodontal examination indices were recorded. A flowchart illustrating the study design and participant recruitment process is presented in **Figure 1a**.

### 2.2 Clinical periodontal examination

A comprehensive full-mouth periodontal examination was performed for all participants by a single calibrated periodontist. Periodontal measurements were recorded at six sites per tooth (mesio-buccal, mid-buccal, disto-buccal, mesio-lingual, mid-lingual, and disto-lingual) using a manual periodontal probe. The recorded clinical parameters included probing depth (PD), sulcus bleeding index (SBI), and the number of teeth lost due to periodontitis.

### 2.3 Subgingival plaque collection and processing

Following the clinical examination, periodontal sites with PD ≥ 5 mm and bleeding on probing (BOP) were identified as target sites. To ensure representative sampling, the site with the deepest periodontal pocket in each quadrant was selected. Subgingival plaque samples were collected using a standardized protocol. Briefly, supragingival plaque was carefully removed with sterile cotton pellets. The site was isolated with cotton rolls and subgingival plaque from the deep periodontal pocket was collected using a sterile Gracey curette, ensuring no contact with saliva or soft tissues. Collected samples were immediately suspended in 1.5 mL of sterile RNase/DNase-free phosphate-buffered saline (PBS) and transported on ice. For each participant, samples from all four quadrants were pooled. The pooled samples were centrifuged at 10,000-16,000 × g for 15 minutes at 4°C. The supernatant was discarded, and the resulting pellets were flash-frozen in liquid nitrogen and stored at -80°C until DNA extraction.

### 2.4 DNA extraction and 16S rRNA gene amplicon sequencing

Total genomic DNA was extracted from the subgingival plaque samples using the CTAB method (Wilson, 2001). DNA concentration and purity were assessed via 1% agarose gel electrophoresis. The V4 hypervariable region of the bacterial 16S rRNA gene was amplified using specific primers: 515F (5’-GTGCCAGCMGCCGCGGTAA-3’) and 806R (5’-GGACTACHVGGGTWTCTAAT-3’) (Caporaso et al., 2011). The PCR reaction mixture (30 µL) contained 15 µL of Phusion^®^ High-Fidelity PCR Master Mix (New England Biolabs, Ipswich, MA, USA), 0.2 µM of each primer, and 10 ng of template DNA. Thermal cycling conditions were as follows: initial denaturation at 98°C for 1 minute, followed by 30 cycles consisting of denaturation at 98°C for 10 seconds, annealing at 50°C for 30 seconds, and extension at 72°C for 30 seconds. A final extension was performed at 72°C for 5 minutes. PCR products were purified using magnetic beads, pooled in equimolar amounts, and visualized via 2% agarose gel electrophoresis using the V-ELUTE Gel Mini Purification Kit (Beijing Zhuangmeng Biotech Co., Ltd., Beijing, China). Paired-end sequencing was performed on the Illumina NovaSeq 6000 platform (Novogene Co., Ltd., Beijing, China).

### 2.5 Bioinformatics and diversity analysis

Raw sequencing reads were processed and quality-filtered using the QIIME 2 pipeline (Bolyen et al., 2019). High-quality sequences were clustered into operational taxonomic units (OTUs) at a 97% similarity threshold, and chimeric sequences were removed. Taxonomic classification was assigned against the SILVA reference database (release 138.1). To account for variations in sequencing depth, the OTU table was rarefied to the minimum sequencing depth across all samples. Alpha diversity indices, including Chao1, Shannon, Simpson, and Pielou’s evenness, were calculated. Beta diversity was evaluated using Bray-Curtis dissimilarity matrices using Principal Coordinate Analysis (PCoA). Differences in microbial community structure between groups were assessed using Analysis of Similarities (ANOSIM).

### 2.6 Statistical analysis

Differential taxonomic analysis between groups was performed using Linear Discriminant Analysis Effect Size (LEfSe), which integrates non-parametric statistical testing with effect size estimation. Given the non-normal distribution and compositional nature of microbial relative abundance data, the factorial Kruskal-Wallis sum-rank test was applied to identify taxa with significantly different abundances between groups. Linear Discriminant Analysis (LDA) was used to estimate the effect size of each differentially abundant taxon, and taxa with an LDA score ≥ 2.0 (*log*_10_ scale) were considered discriminative features.

For clinical data, the normality of data distribution was assessed using the Shapiro-Wilk test. Continuous variables conforming to a normal distribution were presented as mean ± standard deviation (SD) and analyzed using the independent sample T-test, whereas non-normally distributed variables were presented as the median and interquartile range (IQR) and compared using the Mann-Whitney U-test. Spearman’s rank correlation analysis was conducted to evaluate associations between periodontal clinical parameters, glycemic indices, and the relative abundance of subgingival taxa at the species level. All statistical analyses and plots were conducted using Python version 3.11.3. P value < 0.05 was considered statistically significant.

## 3 Results

### 3.1 Study population and clinical characteristics

The demographic and clinical characteristics of the study population are summarized in **Table 1**. A total of 60 subjects were included in the final analysis, and assigned into the well-controlled T2DM group (n = 30) and the systemically healthy Control group (n = 30). There were no significant differences in age or sex distribution between T2DM and Control groups (*P* > 0.05). Importantly, periodontal clinical parameters, including mean PD and mean SBI, were comparable between the groups (*P* > 0.05). This indicates that the severity of periodontal destruction was well-matched at baseline, minimizing disease severity as a confounding factor for microbiome analysis. As anticipated by the study design, glycemic indices differed significantly. The T2DM group exhibited significantly higher levels of Fasting Blood Glucose (FBG) and HbA1c compared to the Control group (*P* < 0.001) (**Table 1**, **Figure 1b**).

**Table 1:** Comparison of demographic, periodontal, and glycemic indices between the well-controlled T2DM group and the systemically healthy control group.

Variable	T2DM group (n=30)	Control group (n=30)	*P*
Age (years)	57.47 ± 11.81	51.93 ± 11.64	0.073
Sex (Female/Male)	12/18	19/11	0.071
Mean Probing Depth (mm)	3.71 ± 0.54	4.03 ± 0.83	0.101
Mean Sulcus Bleeding Index	2.40 ± 0.60	2.64 ± 0.78	0.387
Mean Missing Teeth	1.70 ± 1.42	1.07 ± 1.41	0.049*
FBG (mmol/L)	7.15 ± 1.55	5.13 ± 0.39	<0.001***
HbA1c (%)	6.63 ± 0.69	5.38 ± 0.29	<0.001***
T2DM duration (years)	10.0 (5.75-20.0)	NA	NA
Glucose-lowering medications, n (%)			
Metformin monotherapy	17 (56.7%)	NA	NA
Insulin monotherapy	3 (10.0%)	NA	NA
Metformin + Insulin	7 (23.3%)	NA	NA
Dapagliflozin	2 (6.7%)	NA	NA
Acarbose	1 (3.3%)	NA	NA

Significance levels are indicated as follows: ^∗^*P* < 0.05, ^∗∗∗^*P* < 0.001. NA, Not applicable.

Regarding the specific clinical characteristics of the T2DM group, the median duration of diabetes was 10.0 years (IQR: 5.75-20.0 years). All patients in this group were receiving stable pharmacological treatment for glycemic control. The most prevalent glucose-lowering drug was metformin monotherapy, accounting for 56.7% (n = 17) of the patients. This was followed by a combination therapy of metformin and insulin (23.3%, n = 7) and insulin monotherapy (10.0%, n = 3). A smaller proportion of the patients were managed with other oral antidiabetic agents, including dapagliflozin (6.7%, n = 2) and acarbose (3.3%, n = 1).

### 3.2 Microbial community structure and diversity

**Figure 2:**
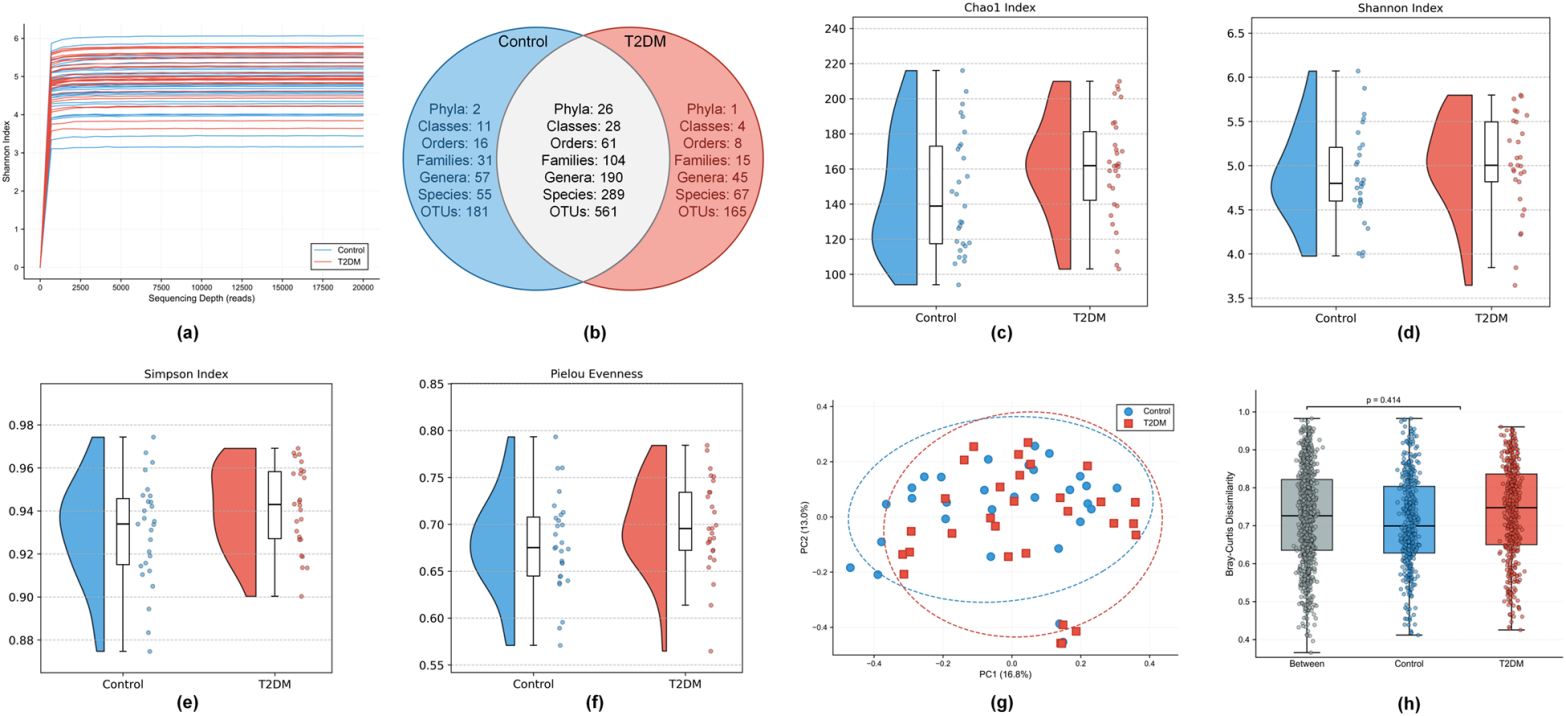
Diversity analysis of the subgingival microbiome in T2DM and Control groups. **(a)** Rarefaction curves based on the Shannon index for all samples. **(b)** Venn diagram displaying the number of shared and unique OTUs (and taxa at different classification levels) between the Control (blue) and T2DM (red) groups. **(c-f)** Raincloud plots comparing Alpha diversity indices between the two groups, including **(c)** Chao1, **(d)** Shannon, **(e)** Simpson, **(f)** Pielou’s evenness. The plots combine boxplots, raw data points, and distribution density. **(g)** Visualization of Beta diversity using Principal Coordinate Analysis (PCoA) based on Bray-Curtis dissimilarity distances. **(h)** Comparison of Bray-Curtis dissimilarity distances within groups and between groups. Statistical significance was evaluated using ANOSIM (*P* = 0.414).

High-throughput 16S rRNA gene amplicon sequencing was performed on microbial DNA isolated from subgingival plaque. After stringent quality control, an average of 82507 ± 20465 paired-end reads were generated per sample. Rarefaction curves reached a plateau (**Figure 2a**), confirming that the sequencing depth was sufficient to capture the vast majority of microbial diversity. Taxonomic annotation identified a total of 907 Operational Taxonomic Units (OTUs), spanning 29 phyla, 43 classes, 85 orders, 150 families, 292 genera, and 411 species. Venn diagram analysis indicated that 561 OTUs were shared between the two groups, while 165 and 181 OTUs were unique to the T2DM and Control groups, respectively (**Figure 2b**).

Regarding overall community structure, the T2DM and Control groups exhibited a high degree of ecological similarity. In terms of alpha diversity, the T2DM group displayed a non-significant increase in species richness compared to the Control group (*P* > 0.05; **Figure 2c**). Consistent with this, no significant differences were observed in composite diversity indices (**Figures 2d, e**) or evenness (**Figure 2f**). Similarly, beta diversity analysis using Principal Coordinate Analysis (PCoA) based on Bray-Curtis dissimilarity revealed no distinct clustering separation, with samples from both groups exhibiting substantial overlap (**Figure 2g**). This observation was statistically confirmed by Analysis of Similarities (ANOSIM), which demonstrated no significant divergence in microbial composition between the two groups (*P* > 0.05) (**Figure 2h**).

### 3.3 Bacterial community structure

**Figure 3:**
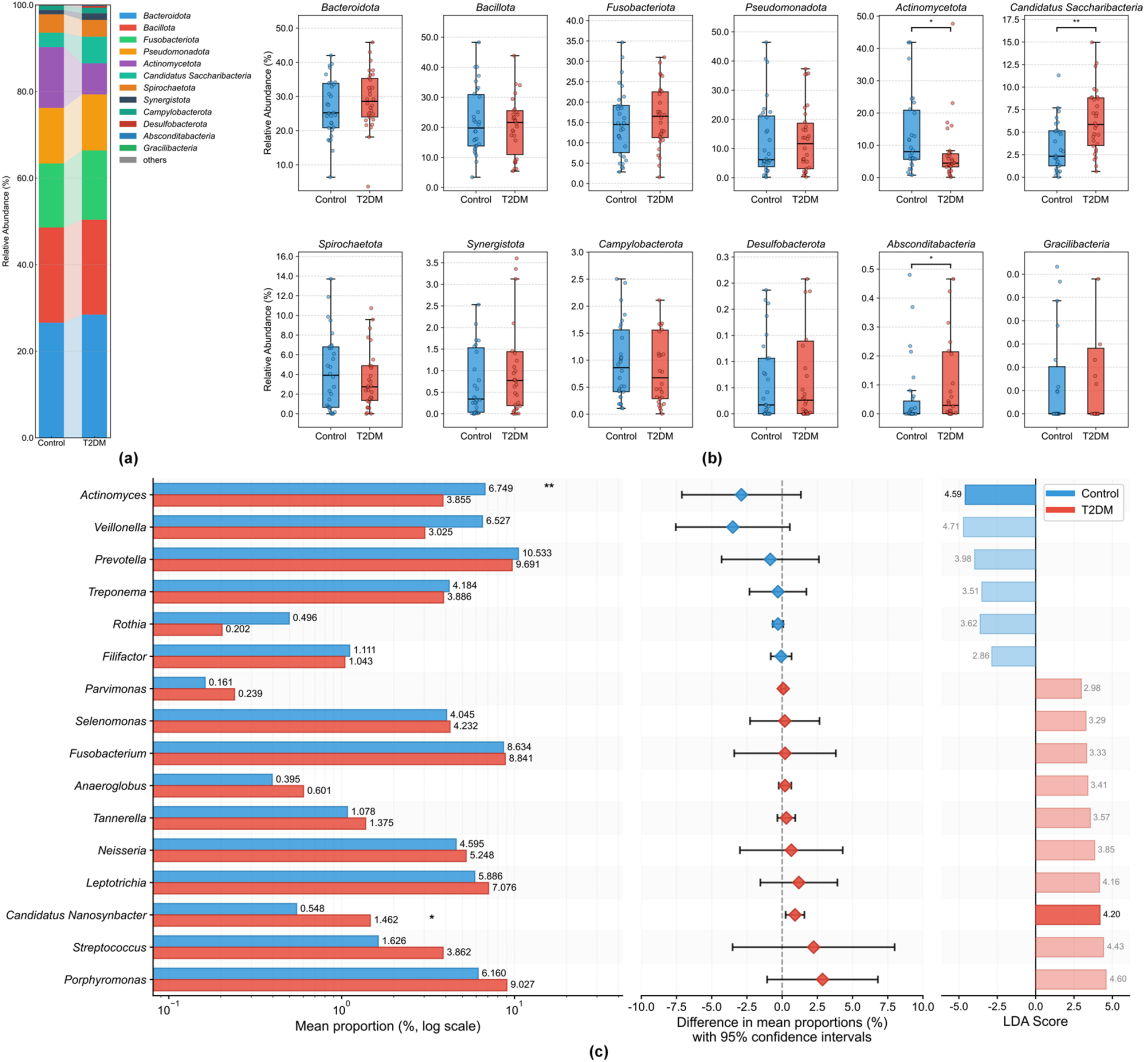
Taxonomic composition and differential abundance analysis of the subgingival microbiome. **(a)** Stacked bar charts showing the relative abundance of the top bacterial phyla in the Control and T2DM groups. **(b)** Boxplots comparing the relative abundance of specific phyla between the two groups. Statistical significance was determined using the Mann-Whitney U test. **(c)** Differential abundance analysis at the genus level. Identification of differentially abundant taxa between Control (blue) and T2DM (red) groups. The left panel shows the mean proportion of taxa (log scale). The middle panel displays the difference in mean proportions with 95% confidence intervals (Extended error bar plot). The right panel shows the LDA scores, indicating the effect size of each differentially abundant taxon. Significance levels are indicated as follows: ^∗^*P* < 0.05, ^∗∗^*P* < 0.01.

At the phylum level, the microbial communities in both T2DM and Control groups were predominantly composed of Bacillota, Actinomycetota, Bacteroidota, Fusobacteriota, and Pseudomonadota (**Figures 3a, b**). The relative abundances of major phyla harboring putative periodontal pathogens, including Bacillota, Bacteroidota, and Fusobacteriota, remained comparable between the groups (*P* > 0.05). Despite the stability in overall diversity, significant differences were observed in some of the less abundant phyla. The most notable alteration was the significant enrichment of the phylum Saccharibacteria (formerly known as TM7) in the T2DM group, where its relative abundance nearly doubled from a mean of 3.34% in controls to 6.30% in T2DM patients (*P* < 0.001). The Absconditabacteria (SR1), which belongs to the Patescibacteria superphylum along with Saccharibacteria, also showed an increase in the T2DM Group (*P* < 0.05). Conversely, Actinomycetota was significantly reduced in the T2DM group (7.33%) compared to controls (14.09%) (*P* < 0.05).

**Figure 4:**
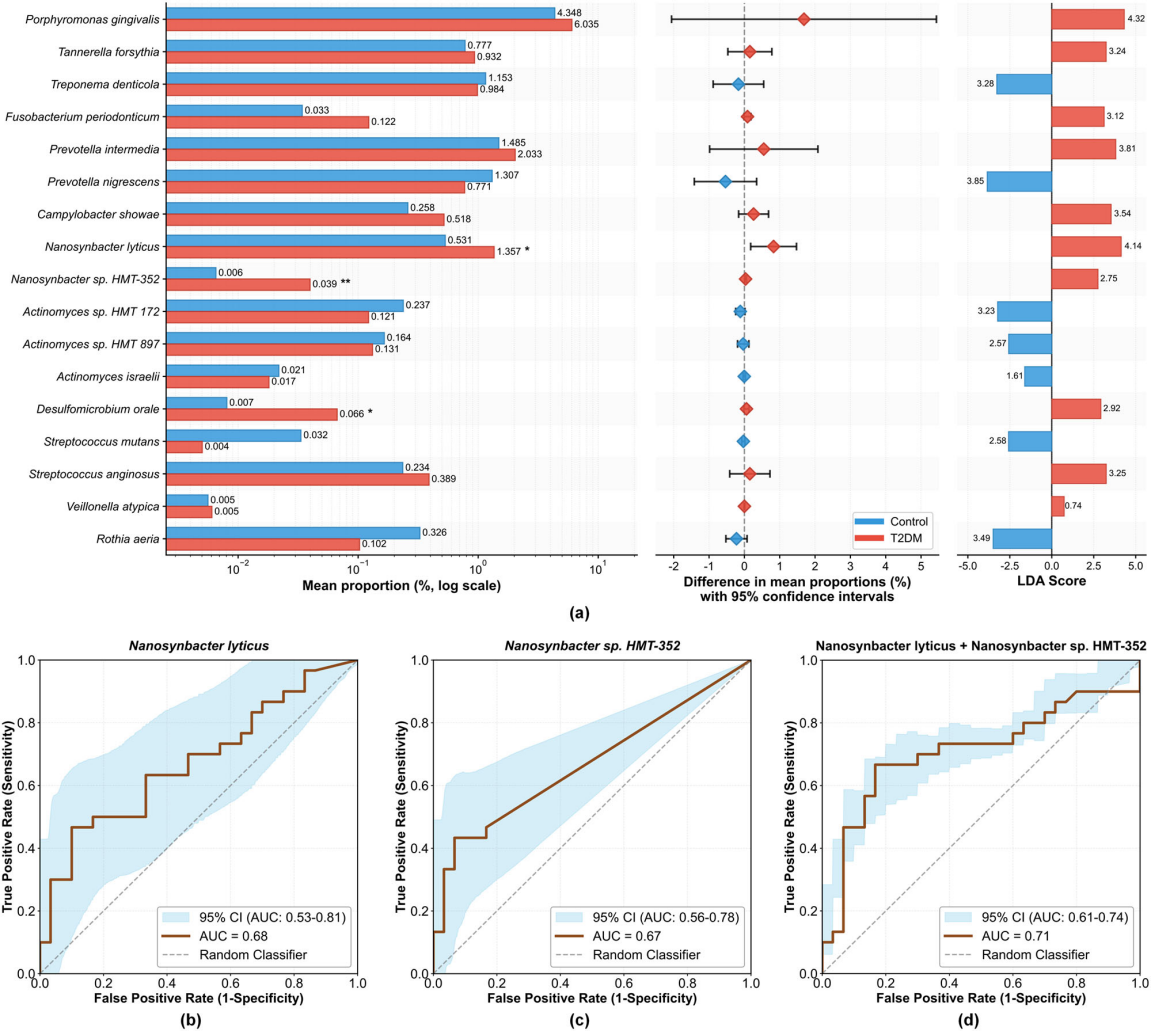
Species-level differential abundance and diagnostic potential of candidate biomarkers. **(a)** Identification of differentially abundant bacterial species between Control (blue) and T2DM (red) groups. The left panel displays the mean relative abundance of each species (log scale). The middle panel shows the difference in mean proportions with 95% confidence intervals (Extended error bar plot). The right panel presents the LDA scores, indicating the effect size of these differences. (b–d) Receiver Operating Characteristic (ROC) curve analyses evaluating the predictive performance of *Nanosynbacter* phylotypes in distinguishing T2DM patients from controls. The plots show the Area Under the Curve (AUC) for **(b)** *Nanosynbacter lyticus* alone (AUC = 0.68), **(c)** *Nanosynbacter* sp.*HMT-352 alone* (AUC = 0.67), and **(d)** the combination of *Nanosynbacter lyticus* and *Nanosynbacter* sp.*HMT-352* (AUC = 0.71). The brown line represents the ROC curve, and the light blue shaded region indicates the 95% confidence interval. Significance levels are indicated as follows: ^∗^*P* < 0.05, ^∗∗^*P* < 0.01.

Subsequent resolution at the genus level elucidated the specific contributors to these shifts (**Figure 3c**). Significant differences were observed in the relative abundance of the genera *Nanosynbacter* and *Actinomyces* between the two groups. Specifically, *Nanosynbacter* levels were significantly elevated in the T2DM group (*P* < 0.05), whereas *Actinomyces* abundance was significantly reduced (*P* < 0.01). This pattern was corroborated at the species level by the significant enrichment of the epibiont *Nanosynbacter lyticus* and *Nanosynbacter* sp.*HMT-352* in the T2DM group (*P* < 0.05; **Figure 4a**). Furthermore, a significant enrichment of the sulfate-reducing bacterium *Desulfomicrobium orale* was observed in the T2DM group (*P* < 0.05). Of particular interest, species-level analysis revealed that the relative abundances of classical periodontal pathogens belonging to the Red Complex (*Porphyromonas gingivalis*, *Tannerella forsythia*, *Treponema denticola*) and the Orange Complex (e.g., *Fusobacterium nucleatum*) did not exhibit statistically significant differences between the T2DM and Control groups (*P* > 0.05) (**Figure 4a**). Receiver Operating Characteristic (ROC) curve was performed to evaluate the discriminative potential of *Nanosynbacter lyticus* and *Nanosynbacter* sp.*HMT-352* (**Figures 4b–d**). The results indicated that *Nanosynbacter lyticus* and *Nanosynbacter* sp. *HMT-352* individually possessed fair predictive value (AUC = 0.68 and 0.67, respectively). Notably, the combined model further enhanced the predictive accuracy (AUC = 0.71), suggesting that combined diagnostic models associated with *Nanosynbacter lyticus* may be more effective in revealing the microbial characteristics of severe periodontitis associated with T2DM.

### 3.4 Correlation analysis of microbiome with clinical indicators

**Figure 5:**
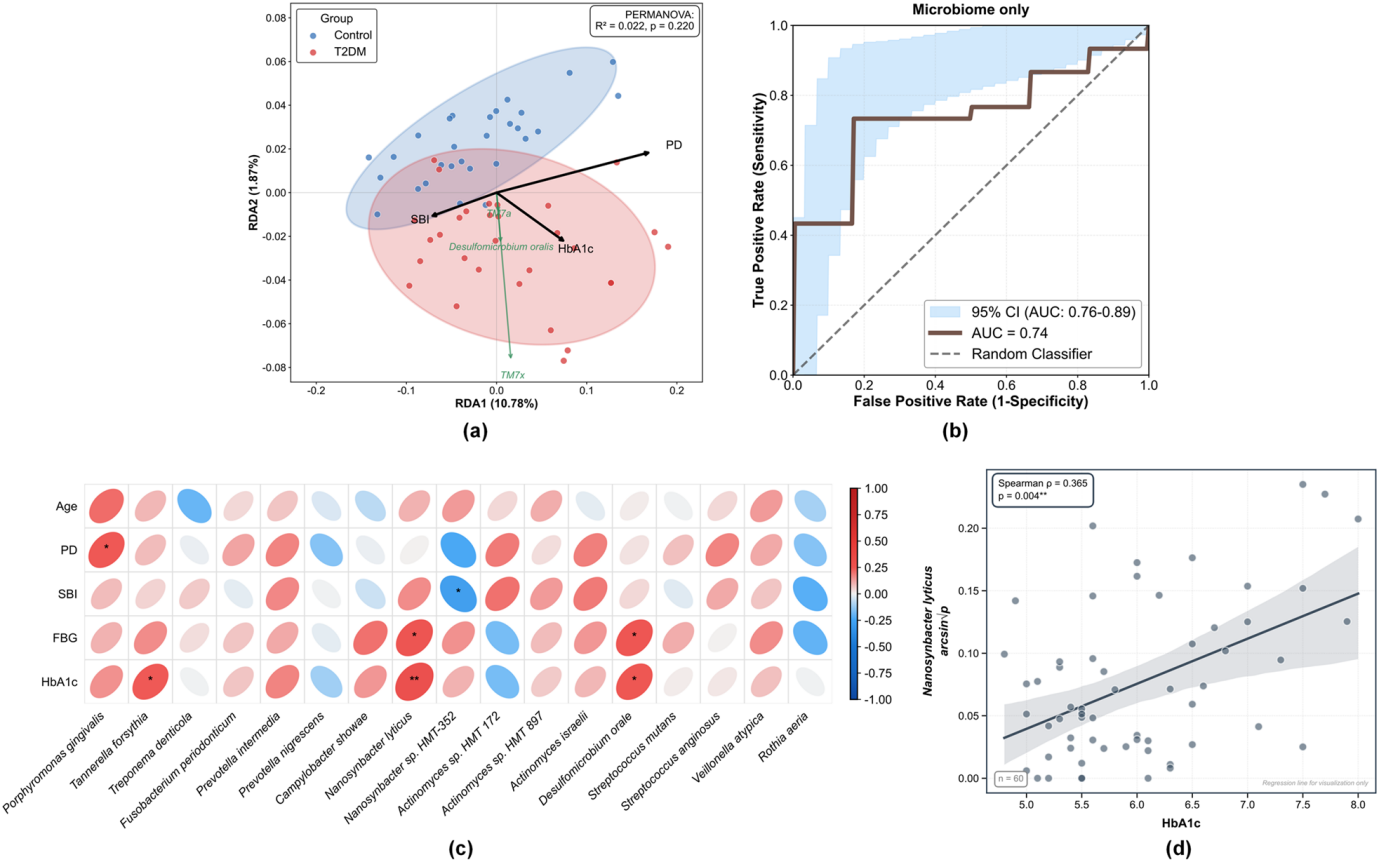
Correlation analysis between clinical parameters and the subgingival microbiome, and predictive modeling. **(a)** Redundancy Analysis (RDA) visualizing the relationship between microbial community structure and clinical environmental factors. Arrows indicate the direction and magnitude of the association for Glycated Hemoglobin (HbA1c), Probing Depth (PD), and Sulcus Bleeding Index (SBI). **(b)** Receiver Operating Characteristic (ROC) curves based on microbial biomarkers for distinguishing T2DM patients (AUC = 0.74). **(c)** Heatmap of Spearman’s rank correlation coefficients between clinical parameters (rows) and key bacterial species (columns). Red ellipses indicate positive correlations, while blue ellipses indicate negative correlations. Significance levels are indicated as follows: ^∗^*P* < 0.05, ^∗∗^*P* < 0.01. **(d)** Scatter plot with linear regression line illustrating the significant positive correlation between *Nanosynbacter lyticus* relative abundance (arcsine-square root transformed) and HbA1c levels (Spearman’s ρ = 0.365, *P* = 0.004).

To explore the link between these microbial alterations and host clinical indicators, Redundancy analysis (RDA) and Spearman correlation analyses were performed (**Figures 5a, c**). The results showed that HbA1c, PD, and SBI were the main environmental factors driving differences in microbial community distribution, and the T2DM group samples clustered along the direction of the environmental factor vector. Reflecting the impact of hyperglycemia on the selection of specific pathobionts, *Nanosynbacter lyticus* showed significant positive correlations with both HbA1c (r = 0.365, *P* = 0.004) and FBG (r = 0.314, *P* = 0.016) (**Figure 5d**). Similarly, the sulfate-reducing bacterium *Desulfomicrobium orale* was positively correlated with HbA1c (r = 0.298, *P* = 0.021) and FBG (r = 0.307, *P* = 0.019). Although *Porphyromonas gingivalis* did not show significant abundance differences between groups, correlation analysis revealed a significant positive association with PD levels (r = 0.283, *P* = 0.031). Conversely, the commensal Actinomyces (e.g., *Actinomyces* sp. *HMT 172*) exhibited a negative correlation trend with glycemic indicators, although this did not reach statistical significance in the current cohort (*P* > 0.05). To further evaluate the clinical utility of these microbial signatures, ROC analysis was conducted. The diagnostic model based solely on microbial biomarkers achieved an AUC of 0.74 (**Figure 5b**). These findings indicate that elevated blood glucose levels are tightly associated with the enrichment of specific microbial taxa.

## 4 Discussion

This study employed the Next-Generation Sequencing (NGS) technique to compare the subgingival microbiome in severe periodontitis patients who are either systemically healthy or with well-controlled T2DM. We discovered that compared to systemically healthy severe periodontitis, T2DM orchestrates a distinct subgingival dysbiosis marked by an increase of the candidate phylum Saccharibacteria concomitant with the reduction of Actinobacteriota.

A major controversy in existing literature is whether T2DM exacerbates periodontitis by increasing the load of classical pathogens (Liu et al., 2018; Qin et al., 2022). We reasoned that the discrepancies may stem from heterogeneous glycemic control and varying periodontal severity in previous cohorts (Matsha et al., 2020; Balmasova et al., 2021). By strictly recruiting patients with Stage III/IV periodontitis and including T2DM subjects with well-controlled blood sugar levels, we observed that the relative abundances of *Porphyromonas gingivalis*, *Tannerella forsythia*, and *Treponema denticola* were comparable between well-controlled T2DM and systemically healthy control groups. Notably, our data revealed between the two groups a significant difference in the relative abundance of those non-conventional bacteria, such as Saccharibacteria and SR1, which have long been considered microbial “dark matter” with limited cultured representatives (Brown et al., 2015; Hug et al., 2016). Particularly, as a member of Candidate Phyla Radiation (CPR), Saccharibacteria are widely distributed in the human oral cavity (Bor et al., 2019; Naud et al., 2023). With a significantly reduced genome and limited metabolic capability, Saccharibacteria exhibit an obligate episymbiotic lifestyle and rely on their host bacteria for survival and propagation (He et al., 2015; Bor et al., 2016). We showed that these ultrasmall episymbiotic bacteria displayed significantly increased relative abundance within the subgingival microbiome in periodontitis individuals with T2DM compared to those with periodontitis but otherwise systemically healthy. Intriguingly, the increase of Saccharibateria and decrease of Actinomyces in relative abundance within subgingival microbiome, a seemingly paradoxical finding considering most of the known host bacteria for Saccharibacteria belong to Actinomyces (Bor et al., 2018, 2020), has been regarded as a signature of periodontal inflammation (Abusleme et al., 2013; Feres et al., 2021). One of the possible explanations is that while there are usually a limited number (1 to 2) of Saccharibacteria cells physically associated with each host bacterium cell, the overgrowth of Saccharibacteria may lead to drastically increased Saccharibacteria to host bacteria ratio, as each host bacterium may harbor over 50 Saccharibacteria cells on its surface (Bor et al., 2018). This could potentially lead to the observed negative correlation between the relative abundance of Saccharibacteria and Actinomyces. Our data suggested that T2DM may exacerbate this negative association between Saccharibacteria and Actinomyces, although the detailed mechanistic understanding of this phenomenon warrants further investigation.

Our study was designed to minimize the noise of uncontrolled hyperglycemia, yet correlation analysis revealed that even within a well-controlled range, glycemic levels remain a potent driver of microbial variance. We observed that the relative abundance of *Nanosynbacter lyticus*, a Saccharibacteria species, was positively correlated with HbA1c, while Actinomyces showed a negative correlation. Mechanistically, this could be driven by metabolic selection. The high glucose levels in the gingival crevicular fluid of diabetic patients lead to an abundance of nutrients within the periodontal pockets (Shim and Babu, 2015; Yilmaz et al., 2018; Rapone et al., 2020). This altered nutrient profile of diabetic gingival crevicular fluid may favor certain organisms, such as Saccharibacteria, and sulfate-reducing bacteria like *Desulfomicrobium orale* (Langendijk et al., 1999), which we also found to be enriched. Specifically, recent studies showed that Saccharibacteria are likely “inflammophilic” (inflammation-loving) bacteria (Hajishengallis, 2014) that thrive in inflammatory nutrition-rich conditions (Chipashvili et al., 2021). While Saccharibacteria have limited metabolic capacity, they can directly uptake and utilize glucose via the glycolysis pathway (Nahar et al., 2026), which could facilitate their growth under glucose-rich microenvironment in T2DM. A recent study investigating the impact of non-surgical periodontal treatment in T2DM patients showed that the reduction in Saccharibacteria abundance was positively correlated with the decrease in HbA1c levels, which offers further, albeit indirect, support for the regulatory role of glycemic control (Xu et al., 2025). Aligning with these metabolic observations, recent genomic insights based on the Human Reference Oral Microbiome (HROM) have identified specific CPR subclades, including Saccharibacteria, as being strongly correlated with periodontitis, capable of synergistically predicting disease alongside *Porphyromonas gingivalis* (Cha et al., 2025). Our study extends the predictive value of Saccharibacteria to the specific context of T2DM. The combined predictive model of *Nanosynbacter lyticus and Nanosynbacter* sp. *HMT-352* enhanced the predictive accuracy (AUC = 0.71). The shifts in the abundance of these *Nanosynbacter* strains indicate a trend of subgingival dysbiosis in patients with T2DM-associated periodontitis, suggesting their potential as adjunctive biomarkers. Furthermore, these microbial biomarkers may offer a more stratified approach, capturing the complex interplay between metabolic status and the oral microbiome to improve periodontal risk assessment.

Several limitations of this study merit consideration. Regarding the study design, the cross-sectional nature of this study precludes establishing causality for the observed inverse relationship between Saccharibacteria and *Actinomyces*. Furthermore, the current data are insufficient to determine whether their potential functional interactions are directly modulated by hyperglycemia. It is noteworthy that the vast majority of patients in the T2DM cohort were taking metformin, which has been shown to affect the host microbiome (Gu et al., 2021). Other confounding factors, such as body mass index, specific dietary habits, and oral hygiene practices, must also be considered. Additionally, to minimize the confounding effects of local inflammation, this study only included patients with severe periodontitis. Consequently, the absence of periodontally healthy control groups limits our ability to characterize the microbial trajectory from health to disease. Methodologically, there are several limitations that warrant consideration. The sampling strategy involved pooling subgingival plaque from the deepest periodontal pocket in each quadrant. While this approach maximizes the detection of anaerobic dysbiosis, it inherently introduces selection bias and may not comprehensively reflect the microbial profiles of shallower diseased sites within the same patient. Moreover, although the calculated sample size in this study is statistically powered to detect shifts in overall community diversity, it may be insufficient to capture subtle differences in low-abundance microbial taxa. Additionally, although taxonomic associations were identified using 16S rRNA gene sequencing of the V4 region, this method cannot fully elucidate strain-level diversity or the active gene expression profiles governing the Saccharibacteria-Actinomyces interaction (Janda and Abbott, 2007). Future longitudinal investigations integrating metagenomic and metatranscriptomic approaches, alongside *in vitro* co-culture experiments, are strictly warranted to validate these biological interplays within a hyperglycemic microenvironment.

## 5 Conclusion

Our findings suggest that periodontitis in well-controlled T2DM patients is associated with a specific dysbiotic signature—marked by enriched Saccharibacteria and reduced Actinomyces—rather than generalized pathogen expansion. This indicates that the diabetic state may uniquely reshape the subgingival niche, persisting even when systemic glycemic levels are managed. Consequently, periodontal therapy for diabetic patients should evolve beyond the traditional “Red Complex” paradigm by prioritizing restoring periodontal eubiosis, including modulating the Saccharibacteria-*Actinomyces* balance to counteract the distinct selective pressures of the diabetic host environment.

## Data Availability

The datasets for this study have been deposited in the NCBI BioProject repository. The data are accessible under the BioProject accession number: PRJNA1429363 (https://www.ncbi.nlm.nih.gov/bioproject/PRJNA1429363).
